# Blood Is Life

**DOI:** 10.4269/ajtmh.20-0961

**Published:** 2021-01

**Authors:** Richard Chimba, Nasreen S. Quadri

**Affiliations:** 1 Selian Lutheran Hospital, Arusha, Tanzania;; 2 Allina Health, Minneapolis, Minnesota

I walk quickly down the corridor toward the laboratory to look for the blood. “Please mister can I get a unit of blood?” I ask. “It is an emergency,” I continue to say with a sense of urgency echoed in my voice. “We have no safe blood. We must wait for the blood bank headquarters to send us the testing results of the samples we requested last week,” the laboratory technician replied. He opens the refrigerator for verification, and I see a quarter of it contains packs of blood labeled by code number. “None of them are safe,” he said. “Before, we used to screen blood here, but it was discouraged by the government because our small laboratory cannot screen efficiently,” he continues to tell me.

As I hear his words, I grind my shoe down hopelessly into the ground beneath me. “What can we do?” I say. Simultaneously, I am thinking about the young 3-year-old boy on the pediatric ward with severe pallor, the palms of his hands as white as a page of paper, weak enough that he cannot even utter a word, and his malnourished status made apparent by his swollen protruding belly. The laboratory technician continues, “Bring his relative to donate blood for replacement. I hope tomorrow we can get results of the samples.” I nod in agreement and add, “Yes, this is safer for the prevention of diseases like hepatitis and HIV, but we need more donors for the blood banks to meet the demand. Our community does not have the habit of donating because maybe they do not know.”

I return back to the young boy’s mother who looks up at me with eyes conveying grief and hopelessness paired with the wrinkles on her face. “He can’t receive blood today, but we may be able to get it tomorrow. In the meantime, call all your relatives to donate blood today,” I uttered sheepishly unsatisfied with my advice. She responds, “I live with my *coco* (grandmother in the Maasai language) and the rest of my relatives live far from here. I could ask my neighbors, but I worry they are intimidated by the ancestral stories of monsters who suck blood.” I too am aware of the widely spread rumors dating back to the 1980s about international Red Cross workers, easily identified by their red colored logo against white cloth, who many people in the community feared were sucking blood and killing people.

That same day, just before sunset, I see three Maasai men accompany the mother toward the laboratory; they are wearing black and red checkered cloth passing under one armpit and tied over the opposite shoulder in typical Maasai fashion. Their faces are still but convey their fear of the anticipated needle drawing their blood and what will happen after they donate blood. One of the men asks one question after another, “Will I be able to stand and walk after I donate the blood? Is it painful? When will my blood recover from donation? Will it cause an increase of excess blood? Will I be able to get the results of the screening on my blood?” Before I can answer, a second man in their group interrupts, “That is why I was refusing to come,” looking both uncomfortable and fearful as his eyes dart around not daring to land in one spot.

I jump in to reassure them, “Don’t worry, it is safe. Most people experience mild pain only. This can be the greatest gift you can give to save a life. Many people die here in Tanzania because of the lack of blood. Please have sympathy and feel the privilege of saving a life. I have personally donated many times for strangers I don’t know while tears drop from my eyes as I think of the impact.” I share with them the reality of rumors and lies about donating blood heard in the community and my personal hopes that 1 day our society will change. The blood donated by these three men, although not directly used to transfuse the young boy, replenished the national blood supply in the context of ongoing shortage. Without adequate voluntary blood donors, personal connections to hospitalized patients in need of blood products is a common reason for individuals in the community to donate blood as family replacement donors even in cases when the patient’s transfusion is not dependent on the replacement.

Thankfully, the next day, the blood bank provided screened blood for the 3-year-old boy hospitalized with anemia. Soon after the young boy received the blood products, I witness the healing power on his mind and inertia in his body. His pale skin and weak limbs transform as his appetite returns, and he smiles happily looking at his mother. Although a common occurrence in our hospitals, this time my heart was touched. I felt the power of this driving force within me. As a clinician who has seen people suffer because of lack of timely blood transfusion, I have a duty to change my peoples’ mind.

The Tanzania National Blood Transfusion Service (NBTS) is a program under the Ministry of Health, Community Development, Elderly and Children, which coordinates the collection, processing, and distribution of blood from voluntary non-remunerated blood donors to all hospitals in the country free of charge. In 1975, the World Health Assembly adopted a resolution, which urged member states like Tanzania to develop comprehensive and coordinated blood transfusion services. Tanzania NBTS has now evolved into a coordinated and centralized system based on voluntary non-remunerated blood donors. The youth in Tanzania account for 80% of the blood supply donors. The blood supply must be both safe and sufficient by undergoing adequate screening for disease prevention and providing adequate supply to those who need it. In Tanzania, the main recipients of blood products are children younger than 5 years (50%), women during pregnancy and postpartum (30%), accident victims, and those undergoing surgery (15%). Since its establishment in 2004, the Tanzanian NBTS has successfully increased blood collection from voluntary non-remunerated blood donors from 52,000 (2006) to 196,735 units (2017). Although this is an excellent increase from prior, total blood collection is still only meeting 86% of the national blood collection target (230,000 units/year). Tanzania also managed to reduce HIV among donated blood from 7.0% (2006) to 1.9% (2017) through improved screening methods.

However, most of my fellow Tanzanians are not aware of the importance of voluntary blood donation. We need to raise awareness in the community on the immense need and tangible benefit of voluntary blood donation. Family replacement donors can be mobilized for voluntary blood donation as their personal connection to a patient in need may inspire benevolence embodied in the action of donation.

Change cannot occur overnight, but every day we see progress in increasing awareness of the benefits of blood donation. Educational campaigns in the community through media can be used to correct misinformation and promote willingness to donate blood and save lives. I have witnessed the impact of gaining trust of community members in the case of my young patient, who made a full recovery. I feel proud to be part of this mission to provide awareness, education, and motivation for my community. We as a country must come together for collective action to increase voluntary blood donation so that one day soon we may achieve 100% of our national blood collection target. This change would impact the morbidity and mortality of those desperately needing life-saving blood products. A simple concept can transform the health of many in our country: blood is life.

